# Muscarinic receptor oligomerization

**DOI:** 10.1016/j.neuropharm.2017.11.023

**Published:** 2018-07-01

**Authors:** Sara Marsango, Richard J. Ward, Elisa Alvarez-Curto, Graeme Milligan

**Affiliations:** Centre for Translational Pharmacology, Institute of Molecular, Cell and Systems Biology, College of Medical, Veterinary and Life Sciences, University of Glasgow, Glasgow G12 8QQ, Scotland, UK

**Keywords:** Muscarinic acetylcholine receptor, Quaternary structure, Dimerization, Oligomerization, Ligand regulation, BRET, bioluminescence resonance energy transfer, CNO, clozapine-N-oxide, EL, extracellular loop, FCS, fluorescence correlation spectroscopy, FRET, fluorescence resonance energy transfer, GMP-PNP, guanosine 5’-[β,γ-imido] triphosphate, htrFRET, homogeneous time-resolved FRET, IL, internal loop, MEU, monomeric equivalent unit, M_1-5_R, muscarinic acetylcholine receptor, NMS, N-methylscopalamine, PI, phosphatidylinositol, QB, quantal brightness, RASSL, Receptor Activated Solely by Synthetic Ligand, RET, resonance energy transfer, RoI, region of interest, SpIDA, Spatial Intensity Distribution Analysis, SR-TPM, spectrally-resolved two-photon microscopy, TIRF, total internal reflection fluorescence microscopy, TM, transmembrane domain

## Abstract

G protein-coupled receptors (GPCRs) have been classically described as monomeric entities that function by binding in a 1:1 stoichiometric ratio to both ligand and downstream signalling proteins. However, in recent years, a growing number of studies has supported the hypothesis that these receptors can interact to form dimers and higher order oligomers although the molecular basis for these interactions, the overall quaternary arrangements and the functional importance of GPCR oligomerization remain topics of intense speculation.

Muscarinic acetylcholine receptors belong to class A of the GPCR family. Each muscarinic receptor subtype has its own particular distribution throughout the central and peripheral nervous systems. In the central nervous system, muscarinic receptors regulate several sensory, cognitive, and motor functions while, in the peripheral nervous system, they are involved in the regulation of heart rate, stimulation of glandular secretion and smooth muscle contraction. Muscarinic acetylcholine receptors have long been used as a model for the study of GPCR structure and function and to address aspects of GPCR dimerization using a broad range of approaches. In this review, the prevailing knowledge regarding the quaternary arrangement for the various muscarinic acetylcholine receptors has been summarized by discussing work ranging from initial results obtained using more traditional biochemical approaches to those generated with more modern biophysical techniques.

This article is part of the Special Issue entitled ‘Neuropharmacology on Muscarinic Receptors’.

## Highlights

• Muscarinic acetylcholine receptors are GPCRs expressed in the nervous systems.
• These receptors can function as monomers and also form dimers and oligomers.
• A broad range of approaches have been employed to study their quaternary structure.
• The main outcomes obtained from over 30 years of work are discussed here.

## 1 Introduction

Dimerization of G protein-coupled receptors (GPCRs), that is the structural arrangement of these receptors in pairs (dimers) at the cellular plasma membrane or within other intracellular membrane structures, and its functional significance remain controversial subjects that have been at the centre of debate for decades. Broadly speaking, at least for class A, rhodopsin-like, receptors the GPCR monomer represents the minimal receptor functional unit (Kuzak et al., 2009, Whorton et al., 2007). However, it is now accepted that receptor dimers, whether homo- or hetero-dimers, not only can be detected in many cells and tissues (Ferré et al., 2014) but may play important roles in receptor ontology and function (Farran, 2017, Franco et al., 2016, Gahbauer and Böckmann, 2016, Margeta-Mitrovic et al., 2000, Milligan, 2004, Milligan, 2009, Milligan, 2013, Smith and Milligan, 2010) as they can display distinct and novel pharmacological features compared to the corresponding monomers.

GPCRs can form not only dimers but also higher-order oligomers where more than two protomers interact as a functional or structural complex, further increasing the complexity of the subject (Marsango et al., 2015a, Navarro et al., 2016, Patowary et al., 2013, Liste et al., 2015). However, one of the caveats of many of the approaches applied to study receptor ‘dimerization’ is an inability of these to resolve and specify whether a detected complex is strictly dimeric or potentially oligomeric. As such the terms ‘dimeric’ and ‘oligomeric’ are often used imprecisely and without intention to specify this feature.

The visual receptor rhodopsin is possibly the clearest example of a class A GPCR demonstrated to be present as a ‘dimer’ in its native setting. Employing atomic force microscopy rhodopsin appears as densely packed rows of pairs of protomers in native mouse disc membranes (Liang et al., 2003, Fotiadis et al., 2006). Although potential caveats in interpretation of these images have been highlighted (Chabre et al., 2003, Suda et al., 2004), such studies provide strong support for the idea that, when in close proximity, the structural organization of the basic 7-transmembrane domain architecture of members of the GPCR family can allow receptor protomers to pack together to allow close association and potential direct physical interactions. This has opened new avenues for studies of receptor function and organization related not only to the molecular structure of potential receptor dimers but also in relation to their interaction with signal transducer proteins including G proteins and arrestins (Ferré et al., 2014, Navarro et al., 2016, Szalai et al., 2012). In certain cases, principally for members of the class C, or glutamate-like, family of GPCRs, homo- or hetero-dimeric organization is a pre-requisite for function (Ferré et al., 2014, Kniazeff et al., 2011, Lane and Canals, 2012, Vafabakhsh et al., 2015). For example, metabotropic GABA_B_ receptors display an absolute requirement for the co-expression of two distinct 7-transmembrane domain polypeptides (GABA_B_ receptor 1 (GABA_B_ R1) and GABA_B_ receptor 2 (GABA_B_ R2)), derived from distinct genes, to form hetero-dimers to allow the complex to reach the cell surface and act as a functional unit (Ng et al., 1999, Kuner et al., 1999). These GPCRs have also been found to show marked disparity in the ligand binding properties of the dimer, depending on the specific two subunits present within the complex. The function of the agonist gabapentin at hetero-dimers formed by distinct splice variants of the GABA_B_R1 (GABA_B_R1a/1b) with the GABA_B_R2 is reportedly very different; in that at GABA_B_R1a/GABA_B_R2 hetero-dimers it acted as an agonist whilst it lacked activity at the GABA_B_R1b/GABA_B_R2 hetero-dimer (Ng et al., 2001). Similar changes in ligand binding and functional properties of hetero-dimers compared to the corresponding GPCR homo-dimers or monomers have also been reported for some class A GPCRs, for example the κ and δ opioid receptors (Jordan and Devi, 1999).

## 2 Muscarinic acetylcholine receptors

The muscarinic acetylcholine receptor family consists of five members (M_1_R-M_5_R) and has long been established as a paradigm for the study of GPCR structure and function, as well as for the development of non-orthosteric receptor ligands. However, the high degree of similarity of the binding pocket for acetylcholine across the family members has hindered the identification of selective orthosteric ligands. As of 2016 multiple crystal structures of four of the receptor subtypes bound by various ligands have been obtained (Haga et al., 2012, Kruse et al., 2012, Thal et al., 2016), leaving only the structure of M_5_R to be determined. Consequently, details of the atomic level structures have begun to be used in structure-based drug design for the identification of subtype selective ligands, whilst also promoting understanding of the mode of binding of various classes of allosteric modulators (Kruse et al., 2014, Miao et al., 2016). Continuing efforts to use such structure-based drug design is resulting in significant advances, as discussed elsewhere in this volume.

Although none of the currently available crystal structures of muscarinic receptor subtypes shows a dimeric arrangement of the receptor, information inferred from the arrangements of the α-helices of the transmembrane domains and potential interaction interfaces identified from both modelling studies and comparisons with atomic level structures of other class A receptors where dimeric contacts have been observed (Geng et al., 2016, Huang et al., 2013, Manglik et al., 2012), have been used to design rational hypotheses for the study of the molecular basis of muscarinic receptor dimerization.

Interestingly, as will be discussed later, studies on both muscarinic M_1_ and M_2_ receptors have suggested that these can present in multiple co-existing and interchanging states, in both transfected model cell systems and in native tissues, with some reports indicating that contacts are fleeting and may be generated by different regions of the receptor structure (Hern et al., 2010, Nenasheva et al., 2013). By contrast other reports suggest that these receptors exist predominantly if not exclusively as dimers (Herrick-Davis et al., 2013) or even as tetramers (Pisterzi et al., 2010, Redka et al., 2013, Redka et al., 2014, Shivnaraine et al., 2016a). Defining this more clearly and assessing why different approaches appear to result in quite distinct conclusions is a key issue for further research on muscarinic receptor (and other GPCRs) dimerization. Finally, a number of studies, both theoretical and experimental, suggest that key interactions between receptor protomers are more likely to be mediated via lipid-based contacts rather than, or in addition to, direct protein-protein interactions (Gupta et al., 2017). Given the long standing interest in muscarinic receptor function and pharmacology, it is hardly surprising that this family of receptors has been used as a model to address aspects of receptor dimerization using a wide range of approaches. In addition to the potential for muscarinic receptor interactions to be intrinsically dynamic, there are recent new insights into the extent to which such interactions can also be regulated by receptor expression levels and by both certain receptor ligands and other receptor-interacting molecules and toxins (Hirschberg and Schimerlik, 1994, Ilien et al., 2009, Alvarez-Curto et al., 2010a, Hern et al., 2010, Hu et al., 2013, Nenasheva et al., 2013, Patowary et al., 2013, Liste et al., 2015, Aslanoglou et al., 2015, Pediani et al., 2016).

This review will examine earlier work and overlay this with results being derived from more recently adopted approaches (see Table 1).

**Table 1:** Summary of approaches used to detect dimers and/or higher-order oligomers of muscarinic receptor subtypes.

Technique	Receptor subtype/model system	Reference
Binding assays	M_2_R; heart tissue	Mattera et al., 1985
	M_2_R; heart tissue	Galper et al., 1987
	M_1_R; brain	Potter et al., 1991
	M_2_R; M_3_R heterologous system	Maggio et al., 1999
	M_2_R; phospholipid vesicles	Redka et al., 2013
	M_2_R; phospholipid vesicles	Redka et al., 2014
Photo-affinity labelling	M_1_R; brain	Avissar et al., 1983
Western blot/Co-Immunoprecipitation	M_3_R; heterologous system	Wreggett and Wells, 1995
	M_3_R; heterologous system	Zeng and Wess, 1999
	M_2_R; heterologous system	Park and Wells, 2004
	M_3_R; heterologous system	Hu et al., 2012
	M_3_R; heterologous system	Hu et al., 2013
	M_3_R; heterologous system	Liste et al., 2015
	M_3_R; heterologous system	Pediani et al., 2016
BRET	M_1_R, M_2_R, M_3_R; heterologous system	Goin and Nathanson, 2006
	M_1_R; heterologous system	Marquer et al., 2010
	M_3_R; heterologous system	McMillin et al., 2011
FRET/htrFRET	M_3_R; heterologous system	Alvarez-Curto et al., 2010a
	M_2_R; heterologous system	Pisterzi et al., 2010
	M_3_R; heterologous system	Patowary et al., 2013
	M_3_R, M_2_R; heterologous system	Aslanoglou et al., 2015
	M_3_R; heterologous system	Liste et al., 2015
TIRF	M_1_R; heterologous system	Hern et al., 2010
	M_2_R; heart tissue and heterologous system	Nenasheva et al., 2013
SpIDA	M_1_R, M_3_R; heterologous system	Pediani et al., 2016
FCS	M_1_R, M_2_R; heterologous system	Herrick-Davis et al., 2013

The first observations suggesting that muscarinic acetylcholine receptors might be arranged in dimers and/or higher-order oligomers were based on results from radioligand binding studies (Potter et al., 1991, Hirschberg and Schimerlik, 1994, Wreggett and Wells, 1995). In the early 1990s, for example, the complex profile of the competition curves between [^3^H]NMS and various agonists to the M_2_R were interpreted as reflecting the presence of two agonist binding sites (guanine nucleotide-sensitive high affinity (H) and low affinity (L) sites) located on dimeric M_2_R molecules in rabbit heart and rat brain stem (Potter et al., 1991). Likewise, computer simulation of the kinetics of binding of the agonist [^3^H]oxotremorine-M at the porcine M_2_R were consistent with the receptor existing as a mixture of monomers and potentially asymmetrical dimers (with one ligand-bound protomer while the second remained unbound) in cultured cells and in porcine atrium (Hirschberg and Schimerlik, 1994). This work also highlighted the impact that levels of receptor expression may have on the equilibrium between monomers and dimers and suggested a degree of cooperativity between protomers in ligand binding (Hirschberg and Schimerlik, 1994). This cooperativity has been further reflected in additional studies on M_2_R where binding data were interpreted in terms of cooperative interactions within receptors organized in higher-order oligomers such as homo-trimers or homo-tetramers (Wreggett and Wells, 1995). This piece of work was also one of the first to show biochemical support for the multimeric nature of the M_2_R, as shown in SDS-polyacrylamide gels of purified receptors from porcine atrial tissue (Wreggett and Wells, 1995). Wells and collaborators have made extensive use of ligand binding studies to gain further insights into the pharmacological profile of M_2_R (Redka et al., 2013, Redka et al., 2014). In competition binding studies, using [^3^H]NMS and seven diverse agonists, these authors observed a dispersion of affinity, indicative of two or more classes of sites (Redka et al., 2013). This has traditionally been explained as the effect of the G protein on an otherwise homogeneous population of sites in studies in which the aggregation state of the M_2_R was not taken into consideration (Birdsall et al., 1978, Ehlert, 1985, Berrie et al., 1979). With this purpose, these authors compared two forms of the purified M_2_R devoid of G protein and reconstituted as a monomer in micellar dispersion or as a tetramer in phospholipidic vesicles (Redka et al., 2013). They concluded that the heterogeneity revealed by the seven agonists at the M_2_R is intrinsic to the receptor tetrameric state, is independent of coupling to G protein and it is, at least in part, a consequence of the cooperativity between linked orthosteric sites (Redka et al., 2013). In subsequent work designed to identify the biologically relevant form of M_2_R, studies compared the ligand binding properties and the effect on the binding profile of the poorly-hydrolysed analogue of GTP, guanosine 5’-[β,γ-imido] triphosphate (GMP-PNP), on reconstituted M_2_R monomers and tetramers, with muscarinic receptors present natively in sarcolemmal membranes (Redka et al., 2014). They concluded that tetrameric but not monomeric forms of the M_2_R resemble muscarinic receptors in such myocardial membranes and suggested that the M_2_R may signal as an oligomer (Redka et al., 2014).

Returning to the early 1990s, in an attempt to study the folding and assembly of GPCRs, Maggio and collaborators (Maggio et al., 1993) generated two hybrid M_3_R/α_2C_-adrenergic receptors in which the first five transmembrane domains (TM) I-V of one receptor were fused to TMVI and VII of the second and *vice-versa* (Maggio et al., 1993). Expression of the individual hybrids was unable to result in stimulation of phosphoinositide (PI) hydrolysis in an agonist-dependent fashion or to allow detection of either adrenergic or muscarinic radioligand binding activity (Maggio et al., 1993). In contrast, co-expression of the two hybrid receptors resulted in the appearance of both muscarinic [^3^H]NMS and adrenergic [^3^H]rauwolscine binding sites and, following incubation of cells co-transfected with the two hybrid receptors the muscarinic agonist carbachol generated an increase in PI hydrolysis (Maggio et al., 1993). Such ‘rescue’ of receptor activity was interpreted to reflect direct interactions between the two hybrid receptors forming a dimeric complex that allowed the reconstitution of functional receptor units (Maggio et al., 1993). Interestingly, co-expression of short hybrid M_3_R/α_2C_-adrenergic receptors in which 196 amino acids were deleted from the internal loop 3 (IL3) prevented the reconstitution of functional receptor units, suggesting a role of the residues located in this internal loop in regulating M_3_R-M_3_R interactions (Maggio et al., 1996).

Although these studies were consistent with the idea of at least a proportion of muscarinic receptors being present as dimers and/or oligomers, they did not provide any intrinsic evidence of a direct physical interaction between protomers. This kind of evidence was obtained sometime later when membrane preparations from rat M_3_R (rM_3_R) expressing cells were analysed by Western blotting under non-reducing conditions (Zeng and Wess, 1999). Such analysis showed several immunoreactive species corresponding in size to putative rM_3_R monomers, dimers and oligomers. Although differential mobility in such gels is challenging to interpret and can reflect protein aggregation stemming from the preparation conditions, subsequent co-immunoprecipitation studies provided further support for the formation of non-covalently associated rM_3_R dimers and oligomers expressed within transfected COS-7 cells and in rat brain membranes (Zeng and Wess, 1999). Moreover, site-directed mutagenesis studies have demonstrated the importance of disulphide-bond formation between conserved cysteine residues located in the extracellular loops (ELs) 2 and 3 of the rM_3_R for protomer-protomer interaction (Zeng and Wess, 1999). Wess and collaborators have made extensive use of Western blot analysis in combination with cysteine substitutions and a disulfide cross-linking strategy to gain insights into mechanisms of muscarinic receptor dimerization (Hu et al., 2012, Hu et al., 2013). Recently, they proposed a model in which rM_3_R-rM_3_R protomers interact to form at least three structurally distinct dimeric species in which protomer-protomer interactions occur as part of the formation of three distinct interfaces. The first proposed dimeric interface, the TMV-TMV interface (Hu et al., 2012), involves residues at the cytosolic end of TMV, the second, the TMIV-TMV-IL2 interface, involves residues in IL2, whilst the third involves residues from the carboxy-terminal Helix VIII and has been designated the TMI-TMII-Helix VIII interface (Hu et al., 2013). Treatment of rM_3_R-expressing COS-7 cell membranes with the muscarinic agonist carbachol was indicated to be without effect on the cross-linking pattern observed using mutants in each of TMV, IL3 or IL2, supporting a hypothesis that TMV-TMV rM_3_R and TMIV-TMV-IL2 rM_3_R dimers form in a constitutive fashion and that these arrangements remain unchanged upon rM_3_R activation. In contrast, agonist-treatment of COS-7 cell membranes expressing rM_3_R-mutants within Helix VIII resulted in an increase in the efficiency of receptor cross-link formation (Hu et al., 2012, Hu et al., 2013).

Although approaches such as immunoblotting, cross-linking and co-immunoprecipitation have been employed to study the basis of GPCR dimerization/oligomerization, they have limitations for the study of interactions involving integral membrane proteins due to the use of non-physiological buffers and detergents that may cause either non-native aggregation or disruption of native biological interactions. Those limitations have been addressed with the development of biophysical methods based on resonance energy transfer (RET) between two molecules, known as the “donor” and “acceptor,” positioned within a restricted distance (in the region of 2–8 nm) and defined orientation (Alvarez-Curto et al., 2010b, Ayoub and Pfleger, 2010, Ayoub, 2016). These include both bioluminescence resonance energy transfer (BRET) and variants of fluorescence resonance energy transfer (FRET), and both have been widely applied to the study of protein-protein interactions and the dimerization of muscarinic receptors and other GPCRs in particular (Goin and Nathanson, 2006, McMillin et al., 2011, Alvarez-Curto et al., 2010a, Ciruela et al., 2010, Marsango et al., 2015a, Sposini et al., 2015).

The most significant difference between these approaches is that BRET measures energy transfer between a bioluminescent donor (most usually variants of the luciferase from *Renilla reniformis*) and a fluorescent acceptor (eYFP, GFP or other) while FRET takes place between two fluorescent proteins with overlapping emission and excitation spectra (of the donor and acceptor, respectively) after the excitation of the donor molecule by an external light source (Ciruela et al., 2010). In both FRET and BRET studies, it is important to experimentally determine that the energy transfer (*E*_(RET)_) between donor- and acceptor-tagged species exceeds the *E*_(RET)_ between the co-expressed and unlinked donor and acceptor molecules, in order to be able to distinguish between specific oligomerization and random collisions. Moreover, the (*E*_(RET)_) between donor- and acceptor-tagged species should be compared to that from donor- and acceptor-linked to known non-interacting proteins.

An example of the use of RET techniques, in combination with molecular studies and site-directed mutagenesis was also provided by Wess and collaborators in a study in which the mechanism of homo-dimerization of the human M_3_R (hM_3_R) was assessed and protomer-protomer interfaces of dimerization mapped (McMillin et al., 2011). Mutants in which selected outward, lipid-facing residues within each of the TMs were simultaneously replaced by alanines were produced. By performing BRET assays using such mutants the authors were able to identify residues in TMs I-V and VII that impaired the ability of these variants to form dimers. The results of this study were interpreted by means of a model in which hM_3_R exists as multiple, energetically favourable, homo-dimers characterized by different geometries and in which protomer-protomer interactions could occur through each of TMV-TMV, TMVI-TMVII, TMIV-TMV and TMI-TMII (McMillin et al., 2011, Fig. 1).

**Fig. 1:**
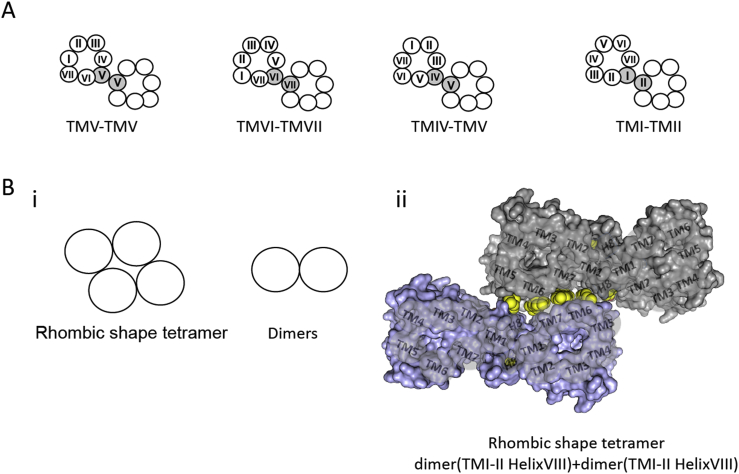
**Quaternary organization of the hM**_**3**_**R.** (**A**) Representation of the four distinct low energy M_3_R dimeric structures as described by McMillin et al., (2011). The transmembrane domains identified as being important for hM_3_R protomer-protomer interactions are shown in *grey circles*. (**Bi)** Schematic representation of the quaternary arrangements of M_3_R as described by Patowary et al., (2013). M_3_R can form rhombic-shaped tetramers and dimers that are in equilibrium at the cell membrane. (**Bii)** Molecular model of the M_3_R tetramer with a rhombic arrangement as a complex of two dimers represented as *grey and blue surfaces*. Predicted molecules of cholesterol are shown as *yellow spheres* (Figure adapted from Liste et al., 2015).

In broad agreement, Patowary et al., (2013) showed that at the cell surface of a HEK293-derived cell line the hM_3_R is able to form not only homo-dimers, but also higher-order oligomers. Herein spectrally-resolved two-photon microscopy (SR-TPM) allowed mathematical fitting of the data to indicate the hM_3_R as being predominantly tetrameric, with the contributing hM_3_R protomers being organized in a rhombus-shaped complex. This tetrameric form was shown to be in equilibrium with dimeric species (Patowary et al., 2013, Fig. 1). This model has subsequently been supported by mutational studies in which outward facing residues of TMI, TMIV, TMV, TMVI, TMVII as well as Helix VIII were replaced with alanines and the ability of such mutants to form dimers assessed using homogeneous time-resolved FRET (htrFRET, see below for further details) (Liste et al., 2015). The mutagenic strategy was based on both the earlier studies described above (McMillin et al., 2011) and molecular modelling studies that took as a starting point a high resolution, inactive state, structure of rM_3_R (Kruse et al., 2012). Although many mutants impaired the competence to the receptor to generate effective interacting complexes, in no case were protomer-protomer interactions fully abolished (Liste et al., 2015). This also suggested the potential of the hM_3_R to form higher-order complexes. To define these complexes, both rhombic (Patowary et al., 2013) and linear (Manglik et al., 2012, Huang et al., 2013) tetramer models were considered, as these were the only ones in which modelling allowed the simultaneous binding of two heterotrimeric G-proteins in their nucleotide-free form, as in the atomic level crystal structure of the β_2_-adrenoceptor complexed with nucleotide-free Gα_s_ (Rasmussen et al., 2011). However, even though both models could explain roles for TMI and Helix VIII as well as TMV and TMVI, only the rhombic-shaped tetramer was compatible with a role of TMVII in a dimer + dimer interface involving TMVI-TMVII and part of TMI (Liste et al., 2015). This model generated a complex of two dimers (in which protomer-protomer interactions occur through an interface involving residues from TMI-TMII and Helix VIII) that interact to form a dimer + dimer interface utilizing residues from TMVI-TMVII and part of TMI. Moreover, molecules of cholesterol were specifically introduced into the model in positions that had already been observed in other published class A GPCR crystal structures (Liste et al., 2015). In particular, two cholesterols interacting with the extracellular side of TMVI (making a total of four molecules in the tetrameric complex) were suggested to form a buffer between the dimers and to mediate interactions of TMVI with TMVII, as well as with residues from TMI (Fig. 1**)**. Molecules of cholesterol in equivalent locations have been described in both the quaternary arrangements of the adenosine A_2A_ (Jaakola et al., 2008) and μ-opioid receptors (Manglik et al., 2012). Furthermore, these cholesterols superimposed well with those observed in the extracellular side of the TMVII of the P2Y12 receptor (Zhang et al., 2014). Two molecules of cholesterol were also described at the TMI- Helix VIII dimer interface positioned as observed in the crystal structure of the β_2_-adrenoceptor (Cherezov et al., 2007) and serotonin 5-HT_2B_ (Wacker et al., 2013) receptors. The organization of the M_2_R has also been investigated using FRET-based approaches (Pisterzi et al., 2010). Herein, as measured using combinations of fluorescence intensity-based microscopy and fluorescence lifetime measurements, and in accord with the ligand binding studies discussed earlier, these studies also concluded that the M_2_R is present as a tetramer at the cell surface of transiently transfected CHO-S cells. Subsequently, a combination of single-particle photobleaching, FRET, dual-color fluorescence correlation and molecular dynamics produced similar conclusions that M_2_R exists as a tetramer, but also suggested that each of the protomers in this arrangement is coupled to a G_i_-family G protein. This conclusion produces a complex of hetero-octamers in which the adjacent protomers interact via an oligomerization interface composed of residues within TMIV and V and in which each of the protomers directly communicates with its coupled G protein and indirectly with the G protein coupled to a neighbouring protomer (Shivnaraine et al., 2016a). In a parallel study, Shivnaraine et al., (2016b) concluded that only interactions between constituent protomers of an M_2_R oligomer complex could explain the observed allosteric effects of ligand binding that are characteristic of M_2_R in myocardial preparations. To monitor such allosteric interactions, the authors developed an M_2_R conformation sensor at the allosteric site, based on FRET between inserted ‘FlAsH’ (Hoffmann et al., 2010) sequences and the mCherry fluorescent protein and performed pharmacological assays involving mutants engineered to preclude intramolecular effects (Shivnaraine et al., 2016b).

Aside from efforts to define dimeric interfaces taking a strictly structural perspective, RET techniques have been widely used to detect muscarinic receptor dimers in living cells. In early studies, Goin and Nathanson (2006) used BRET to demonstrate that each of M_1_R, M_2_R and M_3_R have the ability to form both homo- and hetero-dimers that varied slightly in their interaction affinities and suggested a propensity to form homo-dimers rather than higher-order or hetero-meric complexes. Such BRET studies, however, did not allow discrimination between receptors at the cell surface and the total receptor population present within the cell. Detection of dimerization of the M_3_R has, therefore, been studied in greater detail using FRET-microscopy to allow selection of specific regions of interest, for instance, within the plasma membrane (Alvarez-Curto et al., 2010a, Patowary et al., 2013).

Homogeneous time-resolved FRET (htrFRET), which does not require the use of a microscope, has also been developed and extensively used in the study of GPCR oligomerization (Maurel et al., 2008). Herein, specific self-labelling protein tags e.g. SNAP, CLIP or HALO tags (Alvarez-Curto et al., 2010a, Hussain et al., 2013, Kolberg et al., 2013, Aslanoglou et al., 2015, Marsango et al., 2015a, Marsango et al., 2015b, Ward et al., 2010) have been fused to (usually) the N-terminal domain of a GPCR. Covalent labelling with specific lanthanide (terbium or europium) cryptates that act as energy donor, and a compatible energy acceptor allow htrFRET. Use of non-cell permeant substrates to label the tags allows the exclusive detection of those receptors present at the cell surface. Additionally, the long lifetime of emission from the donor lanthanide means that the signal can be recorded at times after which short-lived cellular autofluorescence has decayed (Maurel et al., 2008, Alvarez-Curto et al., 2010a, Hussain et al., 2013, Kolberg et al., 2013, Aslanoglou et al., 2015, Marsango et al., 2015a, Marsango et al., 2015b, Liste et al., 2015, Ward et al., 2010). As well as basal homo-dimerization of M_3_R and hetero-dimerization of M_2_R/M_3_R at the cell surface (Alvarez-Curto et al., 2010a, Aslanoglou et al., 2015, Liste et al., 2015) potential regulation of homo-dimer and hetero-dimers formation by ligands has also been investigated using such approaches (Alvarez-Curto et al., 2010a, Aslanoglou et al., 2015, Liste et al., 2015). To explore this Alvarez-Curto et al., (2011) used combinations of wild type hM_3_R and a genetically engineered form of this receptor designated as a ‘RASSL’ (Receptor Activated Solely by Synthetic Ligand) mutant (Conklin et al., 2008, Pei et al., 2008, Dong et al., 2010). The hM_3_-RASSL receptor incorporates mutations in TMIII and TMV that render it unable to bind effectively the endogenous ligand, acetylcholine, whilst in parallel it acquired affinity for the synthetic ligand clozapine-N-oxide (CNO), (Alvarez-Curto et al., 2011). Cells expressing both forms of these receptors (hM_3_R + hM_3_-RASSL) that were tagged with appropriate pairs of fluorescent proteins or with SNAP/CLIP tags were used to demonstrate the presence of homo-dimers (Alvarez-Curto et al., 2010a). Here, it was found that treatment with the agonist carbachol significantly reduced the FRET signal whilst treatment with the muscarinic antagonist atropine was without effect, suggesting that, in the presence of the agonist, the complexity of the quaternary structure of the hM_3_R was reduced (Alvarez-Curto et al., 2010a). However, when measurements were focussed exclusively at the cell surface treatment with appropriate selective agonists (carbachol and acetylcholine for the wild type receptor and CNO for the RASSL) the oligomeric structure became more complex (Alvarez-Curto et al., 2010a). Once more, the antagonist atropine was without effect (Alvarez-Curto et al., 2010a, Alvarez-Curto et al., 2011). Whilst these results appear contradictory, conventional “imaging” FRET using pairs of fluorescent proteins monitors receptor proximity throughout the cell whereas htrFRET using the self-labelling protein tags only detected receptors at the cell surface. This may reflect genuine differences in the effects of ligands upon quaternary structure depending upon cellular location but requires further analysis. The use of htrFRET to analyse muscarinic receptor organization has been further exploited to concurrently monitor homo-dimers of hM_3_-RASSL or hM_2_R and hM_3_-RASSL-hM_2_R hetero-dimers in cell co-expressing hM_2_R and hM_3_R (Aslanoglou et al., 2015). Here once more, atropine had no effect on the extent of dimerization, whilst the selective (in this context) hM_2_R agonist, carbachol, caused an increase in level of hM_2_R homo-dimerization and a reduction in the level of hM_2_R-hM_3_-RASSL hetero-dimerization.

Recently, to gain further insights into the dimerization of GPCRs and potential effects of ligand binding Milligan and collaborators (Ward et al., 2015, Ward et al., 2017, Pediani et al., 2016, Marsango et al., 2017) have adopted a biophysical technique, Spatial Intensity Distribution analysis (SpIDA), developed by Wiseman and co-workers (Godin et al., 2011, Godin et al., 2015, Barbeau et al., 2013). This allows the detection of protein-protein interactions with a spatial resolution of 220 nm; a limitation which is overcome by oversampling the laser spot confocal volume and quantifying the excitation illumination volume for membrane oligomerization measurements as a surface as opposed to a 3-dimensional volume (Pediani et al., 2017) Briefly, SpIDA is based upon the analysis of regions of interest (RoIs) selected within laser scanning confocal images of cells expressing the protein of interest tagged with, for example, an appropriate monomeric fluorescent protein (Godin et al., 2011, Godin et al., 2015, Barbeau et al., 2013, Ward et al., 2015, Ward et al., 2017, Pediani et al., 2016, Marsango et al., 2017). Images are then analysed by constructing fluorescence intensity histograms for the pixels within the RoI and then applying super Poissonian distribution curves. From these, both the average quantal brightness (QB) within the RoI and also the mean fluorescent intensity of the fluorescent particles can be calculated (Godin et al., 2011, Godin et al., 2015, Barbeau et al., 2013, Ward et al., 2015, Ward et al., 2017, Pediani et al., 2016, Ward et al., 2017, Marsango et al., 2017).

The normalization of such values for the QB of the fluorescent label alone (expressed in a manner which ensures that it is appropriately located in cells and is in a monomeric state) allows the determination of the quaternary structure of the tagged protein of interest (expressed as monomeric equivalent unit, MEU) and its density (expressed as particles per μm^2^) (Zakrys et al., 2014, Ward et al., 2015, Pediani et al., 2016). Thus, if a suitably tagged GPCR has a QB twice that of the label in a monomeric state, then it is likely to be a dimer.

In various studies in which the protein of interest was labelled with monomeric enhanced Green Fluorescent Protein (mEGFP) for example, the QB of the fluorescent label alone was determined by performing SpIDA measurements on the basolateral membrane of cells expressing a single mEGFP modified at the N-terminal region by incorporation of a palmitoylation + myristoylation consensus sequence (PM-mEGFP), to target the expression of the mEGFP to the plasma membrane or the equivalent forms of mEGFP linked to the C-terminal region of the monomeric, single transmembrane domain protein CD86 (Zakrys et al., 2014, Ward et al., 2015, Pediani et al., 2016, Marsango et al., 2017). For example, the analysis of the full data set obtained with the PM-mEGFP construct showed these to be distributed in Gaussian fashion with an MEU value very close to 1. This indicates that across the range of expression levels achieved, PM-mEGFP was routinely observed as being monomeric and that even at higher levels of expression it was not erroneously identified as being dimeric or oligomeric (Pediani et al., 2016, Marsango et al., 2017).

The first class A GPCR to which this methodology was applied was the serotonin 5HT_2C_ receptor and it was found that the receptor existed as a complex mixture of oligomeric states from monomer to higher-order oligomers, with the most commonly found state being a dimer (Ward et al., 2015). Interestingly, upon treatment with a number of receptor sub-type specific, but chemically distinct antagonists, this state was transformed into a predominantly monomeric one. Importantly for the potential pharmacological and, indeed clinical, relevance of these observations, washout of the drugs resulted in reformation of the original, complex oligomeric state, indicating the reversibility of the ligand effect (Ward et al., 2015).

**Fig. 2:**
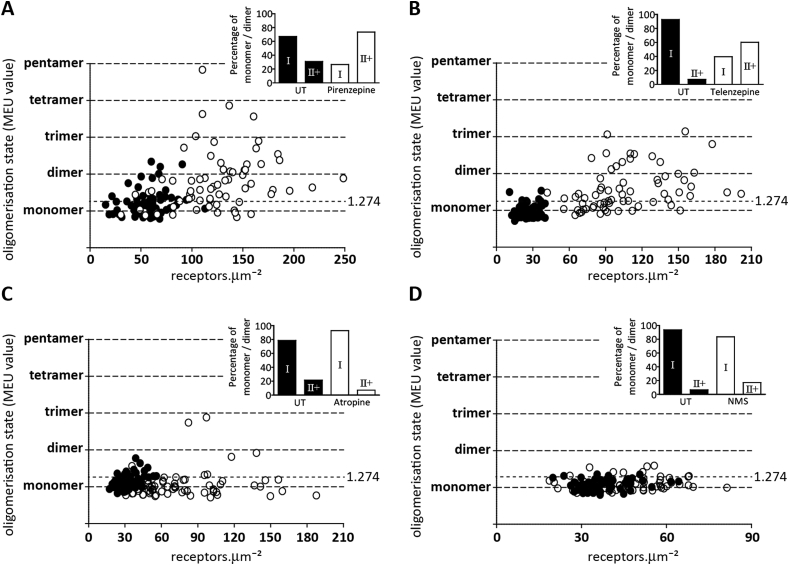
**Pirenzepine and telenzepine alter the quaternary structure of hM**_**1**_**R, whilst atropine and NMS do not.** The quaternary state of the hM_1_R is shown in a graph in which the QB, presented as monomeric equivalent units (MEU), is plotted against the receptor expression level, presented as receptor per μm^2^, in cells not treated (*black circle*) or treated (*open circle*) with pirenzepine (**A**), telenzepine (**B**), atropine (**C**) or NMS (**D**). The percentage of RoIs characterized by the prevalence of hM_1_R in monomeric (QB less than or equal to 1.274 (I)) and dimeric (QB bigger than 1.274 (II+)) state, in not treated (*black bars*) or antagonist treated (*open bars*) cells, is also indicated in the *insert*.

SpIDA has also been applied recently to study the effects of ligands on the quaternary structure of the M_1_R (Pediani et al., 2016). At the basolateral membrane of cells expressing an M_1_R fused to mEGFP, a 75%:25% mixture of M_1_R monomers to M_1_R dimers or higher-order oligomers was detected in the basal state (Pediani et al., 2016). Upon treatment with the M_1_R selective antagonist pirenzepine a large shift from the predominantly monomeric basal state, to a much more complex one containing receptor dimers and oligomers was observed (see Fig. 2). A similar result was also produced by treatment with the chemically closely related M_1_R selective antagonist telenzepine (Pediani et al., 2016). However, this was not a general effect produced by all muscarinic antagonists. For example, neither atropine nor N-methylscopalamine (NMS), produced a change in M_1_R oligomeric structure (Pediani et al., 2016). Furthermore, although markedly selective for M_1_R at higher concentrations both pirenzepine and telenzepine can bind the M_3_R. However, despite both being used at concentrations appropriate to their lower affinity at this receptor, neither antagonist was able to affect the organizational structure of the M_3_R (Pediani et al., 2016). This highlights that ligand regulation may be a receptor selective phenomenon and further studies to understand the molecular differences between M_1_R and M_3_R that result in this divergence should be illuminating. Notably, although not often quoted in reports on muscarinic receptor organization, earlier work by Ilien et al., (2009) had already noted that pirenzepine could promote M_1_R dimerization. These studies indicated that rapid ligand binding to a site on the periphery of the receptor acts as a trigger for a series of conformational changes. These, in turn, were suggested to allow the ligand to access more deeply buried regions of the receptor, promoting the formation of high affinity dimers. An interesting corollary to this is the studies of Hern et al., (2010) who used a single molecule imaging technique, with a resolution of 20 nm, known as total internal reflection fluorescence microscopy (TIRFM) to identify and track in real time individual M_1_R molecules bound to (fluorescent) Cy3B-telenzepine.The receptors were found to be randomly distributed in the outer membrane of transfected CHO cells and at any given time 30% were in the form of dimers, in broad agreement with the work of Pediani et al., (2016).

Hern et al., (2010) considered “dimers” to be those tracks whose intensity was double that of the single fluorophore-ligand non-specifically bound to the glass slide. In more recent studies, the validity of receptor dimerization observed with TIRF analysis has been assessed using SNAP-tagged forms of CD86 (known to be monomeric) and comparing the intensity of its tracks with those measured for SNAP-SNAP-CD86 (Calebiro et al., 2013) or SNAP-CD28 (known to be dimeric) (Tabor et al., 2016).

SpIDA analysis on effects of ligands on muscarinic receptor organization has, to date, centred upon effects of antagonists. This reflects the potential for agonists to promote internalization of the receptor, and the approach requires analysis of receptors located at the cell surface. In the future, use of inhibitors that interfere with clathrin- or dynamin-mediated internalization may be useful. An alternative, and potentially more clear-cut approach, may be to employ genome-edited cells in which receptor internalization is blocked: e.g. using β-arrestin 1/2 knockout HEK293 cells (Alvarez-Curto et al., 2016).

A number of studies have also examined the effects of muscarinic agonists as parts of wider studies on muscarinic oligomerization. For example, Herrick-Davis et al., (2013) made use of Fluorescence Correlation Spectroscopy (FCS) with photon counting histogram analysis to examine the oligomeric structure of a number of class A GPCRs including the M_1_R and M_2_R. These studies concluded that these receptors are exclusively dimeric and that treatment with the agonist carbachol had no effect upon this (Herrick-Davis et al., 2013).

Finally, in a wide range of studies sustained treatment with antagonist ligands has resulted in upregulation of receptor levels and enhanced cell surface delivery. The muscarinic antagonist atropine has been found to increase expression and restore cell surface delivery of many of the mutants that Liste and collaborators generated and that showed the most impaired dimerization/oligomerization characteristics (Liste et al., 2015). Interestingly, long term atropine treatment generally promoted enhanced organization of such mutants, with the majority showing a more similar organization to that of the wild-type receptor (Liste et al., 2015). The role of so called molecular or pharmacological ‘chaperones’ has been widely discussed in the context of receptor trafficking and clearly can promote oligomeric organization at the cell surface. This is likely to be directly linked to early studies that centred on the role of receptor dimerization within the endoplasmic reticulum and the idea of oligomeric contacts as a key quality control points in the ontogeny of many GPCRs.

## 3 Conclusions and future perspectives

In this review, we have summarized current knowledge regarding the quaternary structure of the muscarinic acetylcholine receptor family by considering both of early work, particularly considering outcomes from ligand binding studies, and also results derived from more recently adopted approaches. For at least the M_1_R-M_3_R subtypes, where most work has been focussed, different and sometimes contradictory quaternary arrangements, have been described by various research groups. In this regard, it is important to mention that a large scale comparative study has just been published in which the quaternary structure of 60 class A rhodopsin-like GPCRs was analysed by BRET- and single-molecule microscopy-based assays (Felce et al., 2017). The conclusion was that only a small proportion of class A GPCRs (about 23%) forms authentic dimers while most of them, M_3_R included, are present as monomers in HEK293 cells (Felce et al., 2017).

Moreover, the authors concluded that dimers were formed from closely related phylogenetic clusters and that even closely related receptors could be organized in different quaternary structures (Felce et al., 2017). Finally, the authors hypothesised that dimerization is an evolutionary process, one that increased the “fitness density” of those receptors, such as frizzled and glutamate, for which dimerization is essential for their function preventing them from diverging (Felce et al., 2017). This suggested why dimerization, that does not confer functionality, is not a common future among class A GPCRs (Felce et al., 2017).

Similarly, ligand binding to the receptors has been described as able, or not, to alter the quaternary arrangement of muscarinic receptors. However, despite this, the concept of class A GPCR oligomerization is one which has moved from the periphery of receptor biology to the mainstream. A great deal of extra studies may be required before a coherent picture of the quaternary structure and physiological function of these receptors emerges and it is likely that further studies on muscarinic receptors will be involved in many aspects of this.
